# Chicago Medical Society

**Published:** 1878-09

**Authors:** 


### CHICAGO MEDICAL SOCIETY.

Meeting of August 5, 1878, at the Grand Pacific Hotel. The President, Dr. E. Ingals, in the chair. Fifty members present.

Dr. Tilley exhibited the microphone, illustrating its construction by several instruments. The friction caused by the movement of a camel's hair brush across the frame of the instrument was rendered quite audible to several persons at once, and the ticking of a watch was distinctly heard at several telephones at the same time. Dr. T. expressed the opinion that at present the instrument did not promise any advantage over the instruments already in use for physical diagnosis, corroborating the opinion expressed by Dr. Clement Dufries, of England. Attention was called to a certain resemblance between the instrument and the middle ear, the resemblance being illustrated by a microphone the frame of which was a wooden ointment box. The instrument itself is of the most simple construction, consisting of three small pieces of carbon, held in position by a framework of wood, two of the pieces being attached firmly to the frame work by a wire forming part of an electric circuit being connected with each piece. The third piece is held loosely between the other two pieces, the points of contact being rendered as fine as possible. The battery used was composed of two chloride of ammonium cells, but a more powerful battery would have been more effective. As a receiver of the sound the telephone is necessary.

Under the head of miscellaneous business the following resolutions were presented with reference to an advertisement which had appeared in the daily papers, displaying the names and titles of a number of Chicago physicians.

*Resolved*, 1st, That the Chicago Medical Society considers this advertisement an offense to the honor and dignity of the medical profession.

2d. That the secretary be instructed to notify these physicians that their names are being used in this manner, and request of them an explanation.

After considerable discussion, the resolutions were adopted by an almost unanimous vote.

---

Compliments of the Season.—" The Chicago Medical Journal and Examiner * * always spirited, high-toned and able."—*Edinburgh Medical Journal*, May, 1878.

